# Chronic Inflammation May Enhance Leiomyoma Development by the Involvement of Progenitor Cells

**DOI:** 10.1155/2018/1716246

**Published:** 2018-05-13

**Authors:** Monia Orciani, Miriam Caffarini, Alessandra Biagini, Guendalina Lucarini, Giovanni Delli Carpini, Antonella Berretta, Roberto Di Primio, Andrea Ciavattini

**Affiliations:** ^1^Department of Clinical and Molecular Sciences and Histology, Università Politecnica delle Marche, 60126 Ancona, Italy; ^2^Department of Clinical Science, Università Politecnica delle Marche, 60126 Ancona, Italy; ^3^Clinic of Immunology, Azienda Ospedali Riuniti di Ancona, 60126 Ancona, Italy

## Abstract

Although the etiology of leiomyoma is unclear, a progenitor/undifferentiated cell population has been described whose dysregulation may be involved in the onset of uterine conditions. Moreover, inflammation is involved in the development of several tumors. The aim of this work was to understand if progenitor cells sustain a chronic inflammatory microenvironment that enhances leiomyoma development. Cells from 12 human leiomyoma and 12 normal myometrium samples of the same patients were in vitro isolated and exhaustively characterized (morphology, proliferation, cytofluorometry, differentiation, RT-PCR, immunofluorescence, immunohistochemistry, and Western blotting assays). Selected cytokines (ELISA) and inflammation-related genes (RT-PCR) were analyzed to identify healthy myometrium progenitor cells (MPCs) and leiomyoma progenitor cells (LPCs). Results show that (i) MPCs and LPCs share stemness features, such as immunophenotype and multidifferentiation assay, (ii) LPCs have a significantly shorter doubling time and a significantly higher expression of stemness genes (*p* < 0.05), and (iii) LPCs secreted significantly higher levels (*p* < 0.05) of cytokines related to chronic inflammation and significantly lower amounts (*p* < 0.05) of cytokines related to acute inflammation. Despite the limited sample size, comparisons between leiomyoma and normal myometrium tissue from each patient allowed normalization of patient-specific differences. The evidenced cytokine expression pattern related to chronic inflammation in LPCs may play a role in the increased risk of adverse obstetric outcomes (infertility, spontaneous miscarriage, and preterm birth) in women affected by leiomyomas. These women should be recognized as “high risk” and subjected to specialized management both before and during pregnancy.

## 1. Introduction

Uterine leiomyomas (fibroids) are benign tumors originating from the myometrium and the most common neoplasms of the female reproductive system [1, 2]. They cause prolonged bleeding, pelvic pain, recurrent abortions, and adverse obstetric outcomes and are a significant cause of infertility [3–5]. Their origin and pathophysiology are unclear. A wide range of factors, from genetic aberrations [6] to an undifferentiated cell population that could give rise to them [7, 8], has been investigated. The latter hypothesis is supported by the uterine tissue remodeling that occurs during life in physiological [9] and pathological conditions [10].

One possible explanation for the development of leiomyomas is the dysregulation of mesenchymal stem cell activity [9]. Previous studies [11, 12] have proposed that undifferentiated cells are involved in myometrial pathologies, and also leiomyoma onset may be the result of impaired function, proliferation, and differentiation of undifferentiated cells inside the myometrium that are under the effect of ovarian hormones [13, 14]. Moreover, the clonality of leiomyomas that originate from a single altered cell strongly enforces this hypothesis [1, 15, 16]. Undifferentiated cells have been sought in normal myometrium and leiomyoma tissue by a variety of techniques to address different questions [17–20]. A role for the microenvironment has been suggested for many tumor types, including leiomyoma [21–24], with inflammation appearing to exert a major effect. If the condition causing acute inflammation is not resolved, the inflammation may become chronic, favoring tumor onset and development. Chronic inflammation is maintained by cytokines secreted by the immune system as well as undifferentiated cells [25–29], which are involved in a complex crosstalk with neoplastic cells. These cytokines influence proliferation, fibrosis, and angiogenesis, which in turn sustain fibroid formation and growth [30–32]. Considering that (i) the existence of undifferentiated cells may correlate with leiomyoma onset, (ii) inflammation may sustain leiomyomas, and (iii) cytokines secreted by undifferentiated cells create an inflammatory microenvironment, this study was performed to isolate and characterize undifferentiated cells from myometrium (myometrial progenitor cells, MPCs) and from leiomyoma tissue (leiomyoma progenitor cells, LPCs) and to evaluate the expression of selected inflammation-related cytokines. In addition, the expression of MDR1 (a member of ABC family recognized as a stem cell marker) and of *α*-SMA, collagen type 1, and fibronectin (primary component of the extracellular matrix involved in fibroid development) was tested.

## 2. Materials and Methods

### 2.1. Ethics Statement

All patients provided their written informed consent to participate in the study, which was approved by the institutional ethics committees and was conducted in accordance with the Declaration of Helsinki.

### 2.2. Human Tissue Collection

Leiomyoma and normal myometrium samples were collected from 12 women of childbearing age (range 30–35 years) undergoing hysterectomy for symptomatic fibroids from February to November 2016 at “Salesi Hospital,” Ancona. We investigated normal myometrium and leiomyoma tissue from the same 12 patients with a histologically confirmed diagnosis of leiomyoma. All tissue samples were collected in the operating room under surgical conditions from a trained operator. After removal of the uterus, one small fragment (3–5 mm) from the largest leiomyoma and one (3–5 mm) from normal myometrium was removed by a cold-blade scalpel. The samples were placed in MSCGM medium (Mesenchymal Stem Cell Growth Medium, Lonza, Basel, Switzerland) and sent to our laboratory for processing. We reported the size (in cm), topographic site (anterior, posterior, left lateral, right lateral, and fundal), and location (subserosal, intramural or submucosal) of fibroids from where the samples were obtained. The removal of the sample did not alter the histopathological analysis in any case. All patients displayed good general condition; none of them had a history of myomectomy or uterine surgery, had received medical therapy or oral contraceptives in the previous three months, or had evidence of genital tract infection, endometriosis, or ovarian disease. All had a negative cervical vaginal swab collected prior to surgery, which was performed in the proliferative phase of the cycle. Adenomyosis or other uterine disorders demonstrated on histopathological examination were exclusion criteria.

### 2.3. Cell Culture

Tissue fragments (2-3 mm^3^) were firstly subjected to mechanical digestion then to enzymatic digestion with 0.2% type II collagenase (Sigma-Aldrich, Milan, Italy) at 37°C for 4 hours; subsequently, partially digested solution was placed into 6-well plates containing MSCGM medium which enhances the growth of undifferentiated cells and maintained in culture using same media at 37°C in 95% air and 5% CO_2_. The growth medium was changed after 24 hours to remove unattached cells and then twice a week. Cell morphology was evaluated by phase-contrast microscopy (Leica DM IL; Leica Microsystems GmbH, Wetzlar, Germany) and viability by an automated cell counter (Invitrogen, Milano, Italy). All further analyses involved separate assays of the specimens from each participant up to the first five passages.

### 2.4. Doubling Time

To assess doubling time, 8 × 10^4^ cells/well were plated using an algorithm available online (http://www.doubling-time.com): DT = *t* × lg2/(lgNt − lgN0) where N0 is the number of plated cells, Nt is the number of harvested cells, and *t* is culture time in hours [33].

### 2.5. Characterization of Leiomyoma Progenitor Cells and Myometrium Progenitor Cells

Cells were characterized by testing plastic adherence [34]. Immunophenotype and multipotency were evaluated as previously described [27]. Briefly, for immunophenotyping, 2.5 × 10^5^ cells were stained for 45 min with fluorescein isothiocyanate- (FITC-) conjugated antibodies (Becton Dickinson) against HLA-DR, CD14, CD19, CD34, CD45, CD73, CD90, CD105 OCT4, SOX2, NANOG, and KLF4. Since it is reported [35] that many of the mesenchymal markers are also found in fibroblasts, we analyzed the level of CD9 (Becton Dickinson), which is differently expressed by the two cellular subsets.

For differentiation assay, cells were induced towards osteocytes, chondrocytes, and adipocytes using STEMPRO® Osteogenesis and Chondrogenesis and Adipogenesis Kits (GIBCO, Invitrogen), respectively. Osteogenic differentiation was assessed by von Kossa and alkaline phosphatase (ALP) stainings; adipogenic differentiation was tested by Oil Red staining; for chondrogenesis, cells were cultured in pellet culture system and the sections were exposed to a solution of Safranin-O. Cells cultured in MSCGM alone were used as negative controls.

The expression of stemness genes *(OCT4*, *SOX2*, *NANOG*, and *KLF4)* was analyzed by real-time PCR (RT-PCR) and cytofluorometry as above reported; total RNA was isolated from 1 × 10^6^ cells at passage 4th by using 5 PRIME PerfectPure RNA Purification (5 PRIME, Hamburg, Germany) and retrotranscribed in cDNA (GoScript™ Reverse Transcription System, Promega, Italy). All samples were tested in triplicate with the housekeeping genes RPLP0 and GAPDH for data normalization. Of the two, GAPDH was the most stable and was used for subsequent normalization. After amplification, melting curves were acquired. Direct detection of PCR products was monitored by measuring the fluorescence produced by SYBR Green I dye (EVA Green PCR Master Mix, Bio-Rad) binding to double strand DNA after every cycle. These measurements were then plotted against cycle numbers. The parameter threshold (Ct) was defined as the number of cycles it took to detect a real signal above background fluorescence.

The amount of mRNA detected in LPCs was calculated as X-fold respect to MPCs (expressed as 1) by the 2^−ΔΔCt^ method [33], where ΔCt = Ct (gene of interest) − Ct (housekeeping gene) and Δ (ΔCt) = ΔCt (LPCs) − ΔCt (MPCs). X-fold was calculated for the selected genes in all the twelve samples of LPCs and twelve samples of MPCs. Subsequently, mean ± SD from three independent experiments in triplicates was calculated and displayed. All the primer sequences are reported in Table 1.

### 2.6. Analysis of MDR1 Expression by Western Blotting

MDR1, a member of the large family of ABC transporters, which confer multidrug resistance on human stem cells, was investigated in the two cell types by Western blotting. Briefly, RIPA buffer (150 mM NaCl, 10 mM Tris, pH 7.2, 0.1% SDS, 1.0% Triton X-100, 5 mM EDTA, pH 8.0) containing protease inhibitor cocktail (Roche Applied Science, Indianapolis, IN, USA) was used for protein extraction from 1 × 10^6^ cells at passage 4th. Protein concentration was determined using Bradford reagent (Sigma-Aldrich, Milan, Italy). Total protein extracts (40 *μ*g) were reduced in DTT (0.5 M) for 10 min at 70°C and samples run on a 4–12% gradient precast NuPAGE Bis-Tris polyacrylamide gel for 1 h at 200 V. Electroblotting was performed using iBlot® Dry Blotting System (Invitrogen). Membranes were incubated overnight with primary anti-MRD1 antibody (Santa Cruz Biotechnology, Heidelberg, Germany, 1 : 400) followed by incubation with a secondary antibody conjugated to horseradish peroxidase. Immunoreactive proteins were visualized using a chemiluminescent substrate (Santa Cruz Biotechnology). Anti-*β*-actin (Santa Cruz Biotechnology) was the endogenous control and normal human lung fibroblasts (NHLFs) were the negative control.

### 2.7. Expression of *α*-SMA, Collagen Type 1, and Fibronectin

It is known that even if all mesenchymal stem cells exhibit the original MSC features as defined by the ISCT minimum criteria (spindle-shape, multilineage differentiation, and surface marker expression), the tissue of origin leaves a sort of imprinting in the isolated cells that will express some specific proteins that best characterized their histological source [36–38]. For this reason and to better characterized isolated cells, the expression of *α*-SMA, collagen type 1, and fibronectin expression has been tested by indirect immunofluorescence (IIF) and immunocytochemistry (ICC).

For IIF, 1.5 × 10^4^ cells at passage 3rd were plated, fixed, permeabilized, and treated overnight with mouse anti-human primary antibodies: anti-collagen type I (1 : 1000), anti-cellular fibronectin (1 : 400), and anti-*α*-SMA (1 : 400, all from Sigma-Aldrich, Milano, Italy). Goat anti-mouse FITC-conjugated antibody (Sigma-Aldrich) was the secondary antibody.

Nuclei were visualized using Hoechst 33342 (Sigma-Aldrich, 1 : 1000) under a Zeiss Axiovert 200 M inverted microscope (Carl Zeiss, Jena, Germany).

For ICC, 1.5 × 10^4^ cells from 1 × 10^6^ cells at passage 3rd were plated, fixed, permeabilized, and incubated overnight at 4°C with anti-collagen type I (1 : 1000), anti-cellular fibronectin (1 : 400), and anti-*α*-SMA (1 : 400) monoclonal antibodies. Cells were immunostained using the streptavidin-biotin-peroxidase technique (LSAB universal peroxidase kit, Dako Cytomation, Milano, Italy) and incubated with 3,3-diaminobenzidine. Slides were counterstained with Mayer's hematoxylin.

### 2.8. ELISA and RT-PCR Analysis of the Expression of Inflammation-Related Cytokines

Selected cytokines related to acute and chronic inflammation, IL6, IL12, IFN-*γ*, TNF-*α*, IL2, IL4, IL5, IL13, IL10, TGF-*β*11, IL17A, and G-CSF, were investigated by RT-PCR (as above reported) and by ELISA (Multi-Analyte ELISArray kit, Qiagen, Milan, Italy) as previously described [39]. Briefly, medium conditioned for 72 hours by each sample of MPCs (1 × 10^5^ cells at passage 5th) and LPCs (1 × 10^5^ cells at passage 5th) was used for the test. Samples were dispensed into a 96-well microtiter plate and incubated for 2 hours at room temperature. After washing, avidin-HRP-conjugated antibody was added to the plate and incubated for 30 minutes. Finally, captured cytokines were detected by addition of substrate solution. The OD at 450 nm was determined using a microtiter plate reader (Multiskan GO Microplate Reader, Thermo Scientific).

The levels of the cytokines secreted by leiomyoma cells are reported as a percentage of the levels measured in the corresponding myometrial sample. After, mean ± SD from three independent experiments in triplicates was calculated and displayed. Quantification of mRNA expression in MPCs and LPCs was calculated with the 2^−ΔCt^ method, where ΔCt = Ct (gene of interest) − Ct (housekeeping gene). ΔCt was calculated for the selected genes in all the twelve samples of MSCs. Subsequently, mean ± SD from three independent experiments in triplicates was calculated and displayed.

The expression of other Th1/Th17-related soluble factors (IL22, NFKB1, IL23A, STAT3, CCR5, IL17RA, CX3CL1, CXCL12, and CXCL5) was also assessed by RT-PCR, and the amount of mRNA calculated as above described. All the primer sequences are reported in Table 1.

### 2.9. Statistical Analysis

Statistical analysis of data from at least 3 independent experiments was performed using SPSS 19.0 software (SPSS Inc., Chicago, IL, USA). All data are mean ± SD. For two-sample comparisons, significance was calculated by Student's *t*-test using SPSS 17.0 software. *p* values  ≤  0.05 were considered significant.

## 3. Results

### 3.1. Sample Collection

Twelve 3–5 mm samples of leiomyoma and 12 samples of normal myometrium were collected. The median size of leiomyomas was 5 cm (range 3–8 cm); 3 of them were anterior, 4 posterior, 2 left lateral, 1 right lateral, and 2 were fundal. The location was subserousal in 2 cases, intramural in 4, and submucosal in the remaining six.

### 3.2. Cell Isolation and Characterization

**Figure 1:**
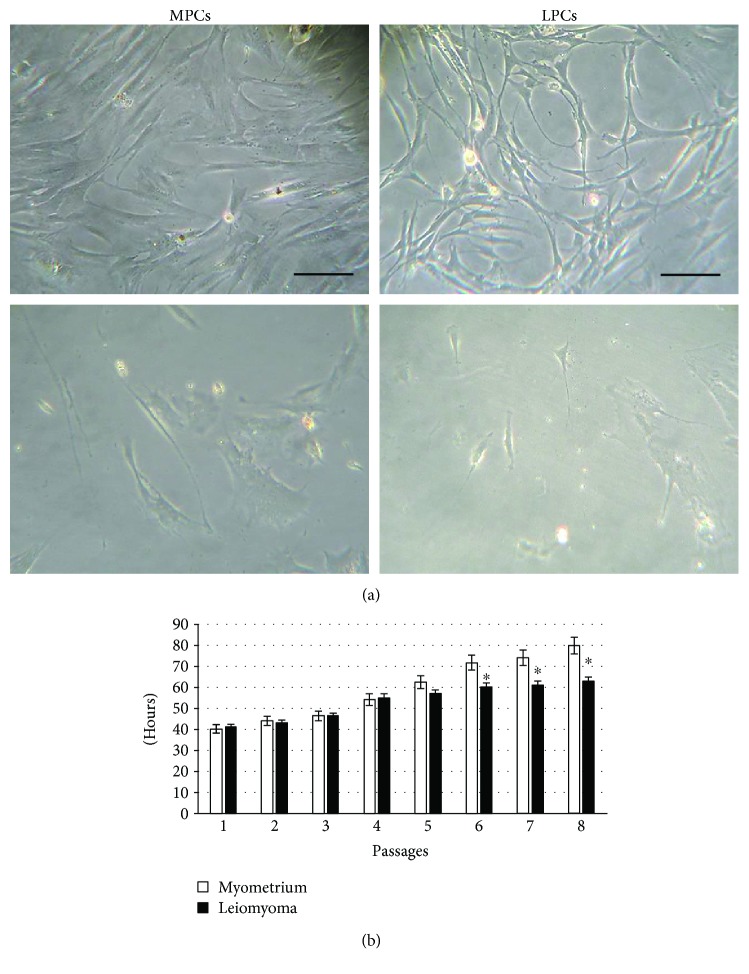
Cell morphology and doubling time. (a) Phase-contrast images of myometrium progenitor cells (MPCs, left) and leiomyoma progenitor cells (LPCs, right) at 2nd (top) and 4th (bottom) passage of culture. Scale bar = 100 *μ*m. (b) Doubling time was calculated over 21 days (8th passage). Data are mean ± SD of experiments performed on 12 samples. ^∗^*p* < 0.05 LPCs versus MPCs.

Leiomyoma and normal myometrium samples from the same 12 patients were used to establish cell cultures. Up to the second passage, the cell population was heterogeneous (Figure 1(a), top panels), probably because it was composed by differentiated and undifferentiated cells; cells displayed different morphologies, from rounded to spindle-like and different sizes. After, cells appeared homogeneous, with a fibroblastoid morphology, (Figure 1(a), bottom panels) and also the cytofluorometric analysis revealed the presence of just one cell population. All subsequent experiments were performed separately on each cell sample. Since no differences were detected among the samples from the two tissue groups, no pairwise analysis was necessary and values are the average of 12 samples.

Doubling time was stable up to the 5th passage and almost identical in the two cell groups; it then increased, the increment being greater in myometrium cells (Figure 1(b)).

Evaluation of the stemness criteria identified by Dominici et al. demonstrated that cells adhered to plastic and that they were strongly positive for CD73, CD90, and CD105 and negative for HLA-DR, CD14, CD19, CD34, CD45, and for the key marker CD9 (Table 2).

**Figure 2:**
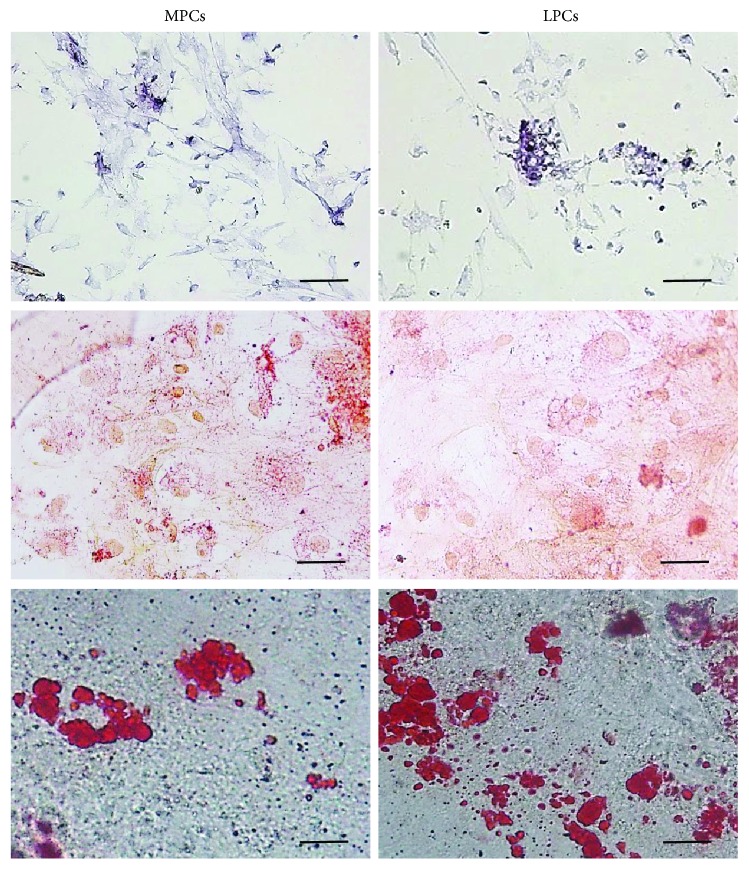
Multilineage differentiation of MPCs and LPCs. Representative images of differentiation experiments. Osteogenic differentiation: ALP staining (top); chondrogenic differentiation: acid mucopolysaccharide coloration with Safranin-O (center); adipocyte differentiation: Oil Red staining (bottom). No differences were noted among different leiomyoma and myometrium samples. Scale bar = 100 *μ*m. MPCs: myometrium progenitor cells; LPCs: leiomyoma progenitor cells.

Cells were also capable of differentiating to osteogenic, chondrogenic, and adipogenic lineages (Figure 2).

**Figure 3:**
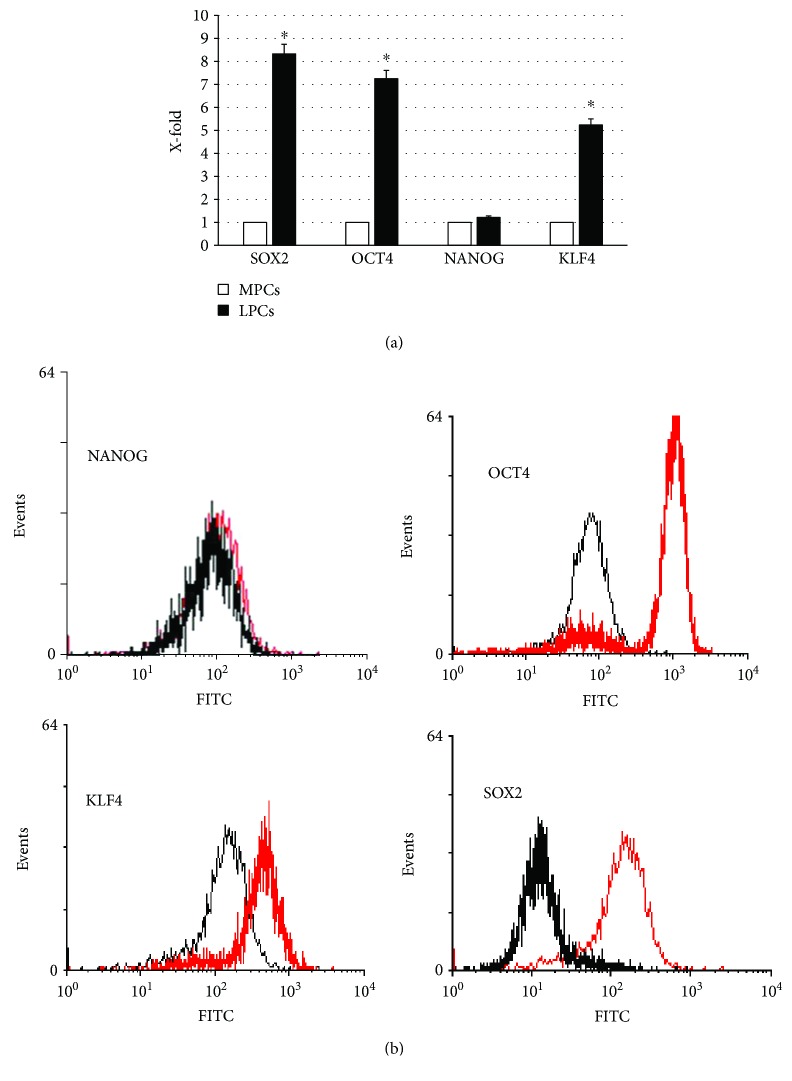
OCT4, SOX2, NANOG, and KLF4 expression. Selected markers of self-renewal and differentiation potential (OCT4, SOX2, NANOG, and KLF4) were evaluated by RT-PCR (a) and cytofluorometry (b). For PCR analysis, the expression levels measured in LPCs are considered as X-fold with respect to MPCs (considered as 1). Data are mean ± SD of analyses performed in 12 different MPC and LPC cultures, upon three independent experiments in triplicates. ^∗^*p* < 0.05 LPCs versus MPCs. For cytofluorometric analysis, representative FACScan analyses of cell-surface antigen expression, as indicated. Black histograms refer to the MPCs and red histograms refer to LPCs.

Both cell types expressed NANGO, OCT4, SOX2, and KLF4, tested by RT-PCR and cytofluorometry, with a higher expression in leiomyoma cells (Figure 3).

Since all experiments confirmed their undifferentiated status, the two cell types were designated, respectively, as myometrium progenitor cells (MPCs) and leiomyoma progenitor cells (LPCs).

### 3.3. MDR1, *α*-SMA, Collagen Type 1, and Fibronectin Expression

**Figure 4:**
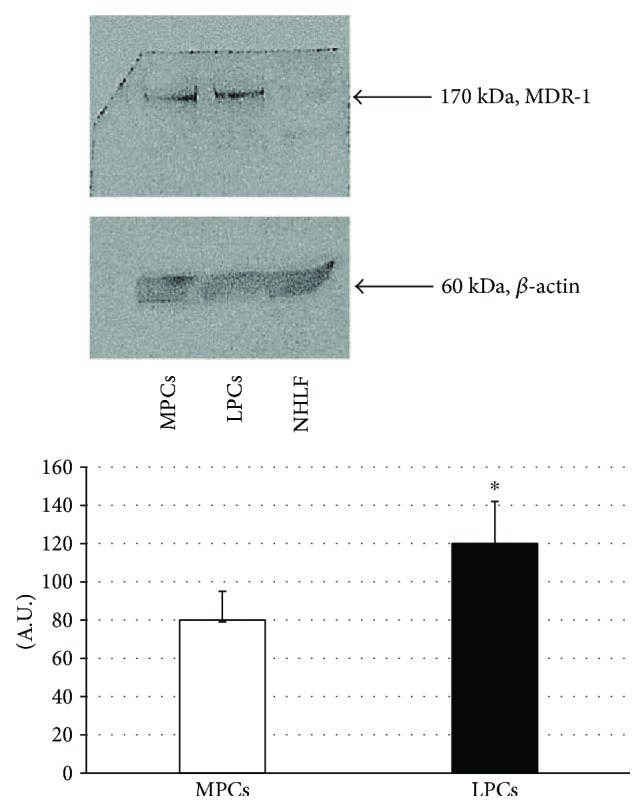
Western blots and densitometric analyses of MDR1 expression. (Top) representative Western blot gels showing the bands of MDR1 and of the endogenous control *β*-actin. (Bottom) densitometric analyses of the immunoreactive bands (quantified as MDR1/*β*-actin bands in corresponding samples and expressed as arbitrary units, A.U.). Data are mean ± SD of analyses performed in MPCs and LPCs from the 12 patients. ^∗^*p* < 0.05 LPCs versus MPCs. MPCs: myometrium progenitor cells; LPCs: leiomyoma progenitor cells.

Western blotting demonstrated a reactive band (molecular weight 170 kDa, corresponding to MDR1) in the MPC and LPC lanes. Densitometric analysis revealed that MDR1expression was higher in LPCs (Figure 4), whereas no signal was detected in NHLFs (negative control).

**Figure 5:**
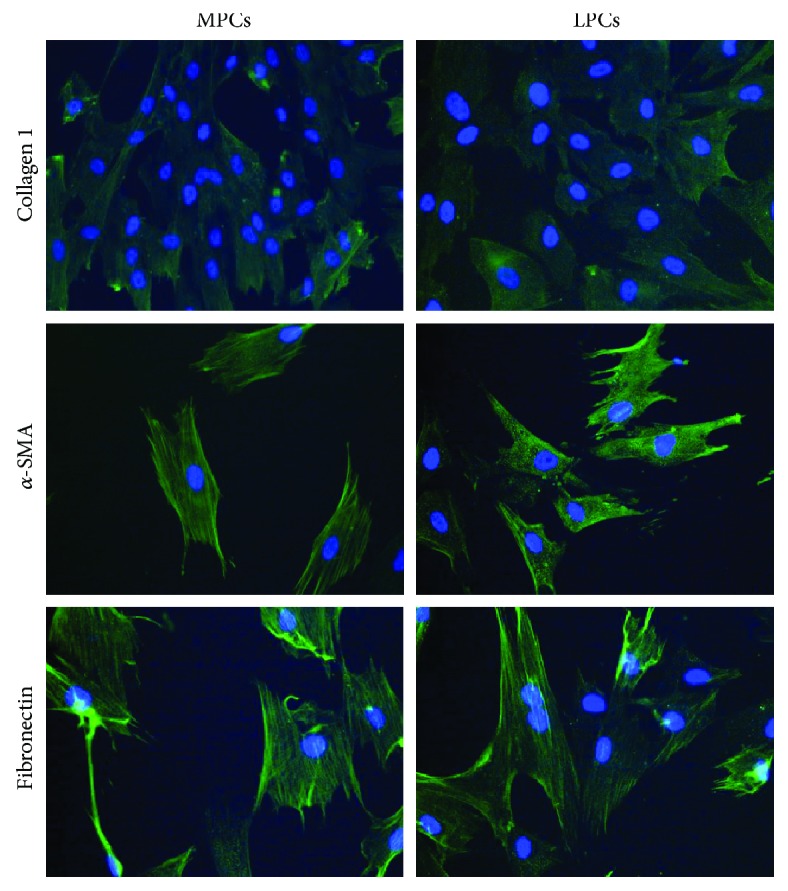
Indirect immunofluorescence analysis of *α*-SMA, collagen type 1, and fibronectin. A secondary FITC-conjugated antibody was used after incubation with the primary antibodies. Nuclei were counterstained with Hoechst 33342. Myometrium progenitor cells (MPCs) and leiomyoma progenitor cells (LPCs) showed a similarly strong positivity for *α*-SMA and fibronectin, whereas collagen type 1 expression was fainter. Differences between MPCs and LPCs were not significant (×200 original magnification).

**Figure 6:**
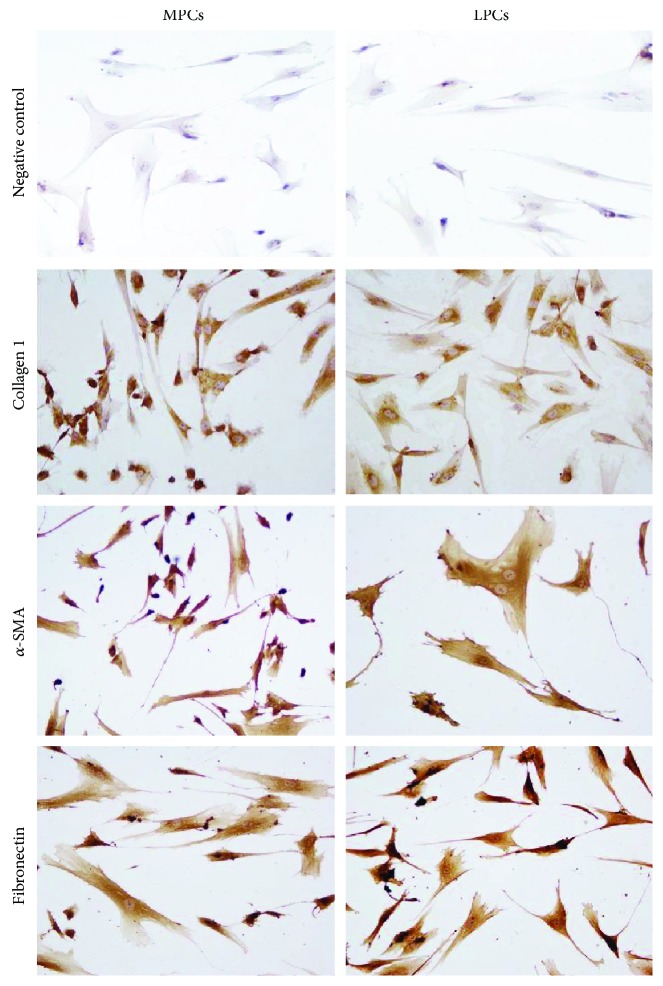
Immunocytochemical analysis of *α*-SMA, collagen type 1, and fibronectin. Compared to the negative control (secondary antibody alone), the primary antibodies induce brownish staining in myometrium progenitor cells (MPCs) and leiomyoma progenitor cells (LPCs). The reaction was weaker for collagen type 1 than for *α*-SMA and fibronectin. Differences between MPCs and LPCs were not significant (immunoperoxidase, ×400 original magnification).

MPCs and LPCs were positive for *α*-SMA, collagen type 1, and fibronectin on IIF (Figure 5) and IIC (Figure 6), without significant differences between the two cell types. Although more than 90% of MPCs and LPCs were strongly positive for all three proteins, the staining for collagen type 1 was fainter than the other two.

### 3.4. Expression Profile of Inflammatory Cytokines

**Figure 7:**
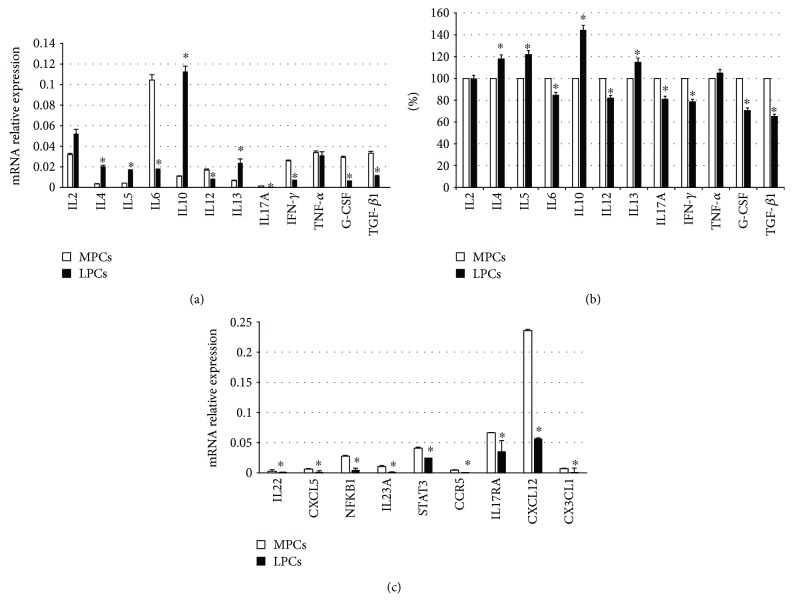
Expression of selected cytokines in myometrium progenitor cells (MPCs) and leiomyoma progenitor cells (LPCs). (a) Quantification of mRNA expression in MPCs and LPCs was calculated with the 2^−ΔCt^ method, where ΔCt = Ct (gene of interest) − Ct (housekeeping gene). ΔCt was calculated for the selected genes on 12 different cultures. Subsequently, mean ± SD from three independent experiments in triplicates was calculated for LPCs and displayed. ^∗^*p* < 0.05 LPCs versus MPCs. (b) ELISA test; the levels measured in MPCs were considered as 100% and those detected in LPCs accordingly calculated; ^∗^*p* < 0.05 LPCs versus MPCs. (c) Selected Th1/Th17 pathway molecules evaluated by RT-PCR. Quantification of mRNA expression in MPCs and LPCs was calculated with the 2^−ΔCt^ method, where ΔCt = Ct (gene of interest) − Ct (housekeeping gene). ΔCt was calculated for the selected genes on 12 different cultures. Subsequently, mean ± SD from three independent experiments in triplicates was calculated for LPCs and displayed. ^∗^*p* < 0.05.

The expression and secretion of inflammation-related cytokines were, respectively, evaluated by RT-PCR (Figure 7(a)) and ELISA (Figure 7(b)).

Compared to MPCs, LPCs exhibited significantly (*p* < 0.05) higher levels of Th2 pathway cytokines (IL4, IL5, IL10, and IL13), with IL10 showing a 40% increase, and significantly (*p* < 0.05) lower levels of Th1/Th17 cytokines (IL6, IL12, IL17A, IFN-*γ*, G-CSF, and TGF-*β*1).

Finally, IL2 and TNF-*α* expression was not significantly different between MPCs and LPCs. Since both mRNA levels and ELISA revealed a strong downregulation of Th1/Th17 pathway cytokines in leiomyoma, the expression of other soluble factors belonging to these pathways was assessed by RT-PCR and found to be lower in LPCs (Figure 7(c)).

## 4. Discussion

Uterine leiomyomas are highly common lesions of unclear etiology. Several hypotheses have been formulated and predisposing factors have been described [40]. Investigation of the factors responsible for the significant plasticity of the uterus has led to the identification of a progenitor/undifferentiated cell population, prompting the hypothesis that its dysregulation may be implicated in the development of uterine pathologies [8, 41, 42]. Various approaches have been applied to identify and characterize progenitor cells [17–20, 43].

Since inflammation is a recognized mechanism underlying the onset of several tumors, the role of an inflammatory microenvironment has also been explored in leiomyoma development. The overall hypothesis is that leiomyomas are caused in part by an immune milieu that is chronically inflammatory [28]. In addition, the chronic inflammatory state increases estrogen which in turn may increase leiomyoma growth [44]. Chronic inflammation is sustained by specific cytokines secreted by immune, undifferentiated, and tumor cells [25, 26] and seems to be exploited by tumor cells to escape the host immune system [25]. Undifferentiated cells play a central role in the microenvironment and modulate the cellular functions of a variety of immune cells including B and T lymphocytes, natural killer cells, monocytes, and dendritic cells [45–50]. Presumably, this role is operated by a complex interplay of short- and long-range signaling that may entail a wide spectrum of molecular mediators, including soluble cytokines and growth factors [51].

However, a correlation between undifferentiated cells and inflammation in leiomyoma onset has never been explored. In the present work, the issue was investigated through isolation and extensive characterization of undifferentiated progenitor cells from 12 specimens of normal myometrium and 12 leiomyoma samples. Demonstration of a stem-like immunophenotype and of the ability to differentiate into osteoblasts, chondrocytes, and adipocytes enabled their designation as MPCs and LPCs. For the first 5 passages, MPCs and LPCs showed a stable and comparable DT; subsequently, the doubling time increased. This increment was higher in MPCs than in LPCs (75.36 ± 4.19 versus 61.55 ± 1.32 hours, resp.). The DT increase corresponded with a reduction in proliferation, which in cultured cells is a sign of senescence; since senescence is greater in more differentiated cells, LPCs seemed to be less differentiated than MPCs. Although this finding disagrees with those of Chang et al. [16], it is consistent with the higher expression by LPCs of stemness genes *(SOX2*, *OCT4*, and *KLF4)* and of MDR1 (as demonstrated by Western blot and densitometric analysis). MDR1 is a member of the ABC transporter family, which is believed to protect stem cells from genetic damage by naturally occurring xenobiotics [52, 53]. ABC family members are considered as stem cell markers and may be used for stem cell purification; treating cells with specific dyes (Rhodamine123 or Hoechst 33342), stem cells show a reduced retention by the presence of this transmembrane proteins capable of pumping these dyes out of the cell [54]. Different roles have been attributed to MDR1, such as drug efflux and protection of cells against apoptotic cell death induced by a variety of causes, and to modulate signal transduction pathways enhancing cell survival [54].

Progenitor cells were further characterized by IIF and ICC through the expression of *α*-SMA, collagen type 1, and fibronectin. Their expression was strong and similar in MPCs and LPCs, although staining for collagen type 1 was weaker. It is now well accepted that progenitor/mesenchymal cells are a very heterogeneous reservoir of cells; even if cells satisfied all the three essential criteria identified by Dominici, progenitor displays biologic properties that may differ according to the tissue of derivation. Specific molecules, receptors that characterized a particular tissue, may be expressed by undifferentiated cells derived from it. In this case, myometrium is characterized by abundant amounts of *α*-SMA, collagen type 1, and fibronectin. We found a detectable expression of these three molecules at mRNA level; interestingly, progenitors from leiomyoma do not hyperexpress these factors compared to cells from myometrium even if it is known their involvement in fibroid development. This apparent contradiction may reside in the fact that accumulation of extracellular matrix (ECM) in leiomyoma may be the result of a dysregulated proliferation of cells; in fibroids, ECM is more abundant because of a more elevated number of producing cells. In vitro experiments were performed using the same amount of cells derived from myometrium and leiomyoma [55]. The expression of collagen type 1 was weaker than the other two molecules; it may depend by the low secretion of TGF-*β*1 observed by ELISA test. TGF-*β*1 is in fact known as a great promoter of collagen type 1 production [56, 57].

As regards the role of inflammation, it is well accepted that leiomyoma onset may correlate with active inflammation [21] and that undifferentiated cells participate in microenvironment formation. For this reason, a panel of 12 cytokines related to acute and chronic inflammation was evaluated in LPCs and MPCs as mRNA expression and secretion.

Although IL6, TNF-*α*, IFN-*γ*, G-CSF, and TGF-*β*1, which have been implicated in leiomyoma development [58, 59], were expressed in both cell types, the most notable finding was the significantly different expression of Th2 and Th1/Th17 pathways in LPCs and MPCs. Indeed, LPCs exhibited a significantly greater expression of IL4, IL5, IL10, and IL13 (Th2 pathway) and a significantly lower expression of Th1/Th17 pathway cytokines. In particular, they secreted less TGF-*β*1 which, alone or combined with IL6, is involved in the differentiation of naive T-cells to Treg T-cells and Th17 T-cells, which in turn secrete TGF-*β*1 and IL17. Treg T-cells are actively involved in inhibiting tissue inflammation, and their suppression may enhance the maintenance of the inflammatory microenvironment that favors leiomyoma development. IL12 and IFN-*γ*, which allow differentiation of naive cells to Th1 T-cells, were lower in LPCs, whereas secretion of IL4, IL5, and IL13, which drive the differentiation to Th2 T-cells, was lower in MPCs; this also applied to IL10, which is subsequently produced.

To lend support to the downregulation of Th1/Th17 pathway cytokines in LPCs, other soluble factors of the same subgroups (IL22, NFKB, IL23A, STAT3, CCR5, IL17A, IL17RA, CXCL12, CX3CL1, and CXCL5) were evaluated by RT-PCR. This panel of molecules with different functions (chemokines, cytokines, transcription factors, and signaling pathway molecules) provided a general picture of the involvement of Th1/Th17 pathways. All molecules were downregulated in LPCs, confirming the upregulation of the Th2 profile. Th2 cells and cytokines are associated with chronic inflammation, whereas the Th1/Th17 pathways are related to acute inflammation. The upregulation of the Th2 pathway in LPCs may reflect a protracted inflammatory state that is maintained by paracrine effect exerted also by undifferentiated cells, which create a stroma favoring leiomyoma development.

These observations suggest a relationship between chronic myometrial inflammation and uterine leiomyomatosis, infertility, and adverse obstetric outcomes [60]. Indeed, a chronic inflammatory reaction induced by fibroids and altered myometrium contractility may hinder embryo implantation, affecting fertility [61–63]. Among the mechanisms invoked to explain the increased myometrial contractility are an excess of cytokines, growth factors, neurotensin, neuropeptides, enkephalin, oxytocin modulators, and chronic inflammation of the fibroid capsule [64–66].

Alterations in the endometrial-myometrial junction (EMJ) seem to play a key role in implantation failure and recurrent miscarriage. The EMJ, the inner third of the myometrium adjacent to the endometrium, provides macrophages and uterine natural killer cells, which are essential for endometrial decidualization in the midluteal window of implantation [67]. It is conceivable that intramural/submucosal fibroids not only physically disrupt the EMJ [68–70] but also cause chronic inflammation, steroid receptor alterations, and ultimately implantation failure. A chronic proinflammatory effect exerted by leiomyoma progenitor cells may explain why even small myomas or early-stage diffuse leiomyomatosis hamper embryo implantation. Inflammatory stimuli also seem to alter progesterone receptor activation; hence, transrepressive activity in myometrial cells, providing support for the hypothesis that tissue inflammation, may be involved in miscarriage and preterm delivery [71].

In conclusion, the present data suggest that (i) progenitor cells are found both in leiomyomas and normal myometrium, (ii) these progenitors show a differential expression of cytokines related to acute and chronic inflammation, and (iii) the upregulation of cytokines related to chronic inflammation in leiomyoma progenitors may favor the formation of a microenvironment suitable for leiomyoma onset and development.

## Figures and Tables

**Table 1:** Primer sequences.

Gene	Primers
GAPDH	Forward 5′-CCCTTCATTGACCTCAACTACATG-3′ Reverse 5′-TGGGATTTCCATTGATGACAAGC-3′

RPLP0	Forward 5′-CCATTCTATCATCAACGGGTACAA-3′ Reverse 5′-TCAGCAAGTGGGAAGGTGTAATC-3′

NANOG	Forward 5′-TGAACCTCAGCTACAAACAG-3′ Reverse 5′-CTGGATGTTCTGGGTCTGGT-3′

SOX2	Forward 5′-ACACCAATCCCATCCACACT-3′ Reverse 5′-GCAAACTTCCTGCAAAGCTC-3′

OCT4	Forward 5′-AGCGAACCAGTATCGAGAAC-3′ Reverse 5-′TTACAGAACCACACTCGGAC-3′

KLF4	Forward 5′-CCCACACAGGTGAGAAACCT-3′ Reverse 5′-ATGTGTAAGGCGAGGTGGTC-3′

IL-17A	Forward 5′-GGTCAACCTCAAAGTCTTTAACTC-3′ Reverse 5′-TTAAAAATGCAAGTAAGTTTGCTG-3′

IL2	Forward 5′-TCACCAGGATGCTCACATTTAAGT-3′ Reverse 5′-GAGGTTTGAGTTCTTCTTCTAGACACTGA-3′

IL4	Forward 5′-GAAGAGAGGTGCTGATTG-3′ Reverse 5′-GGAAGAACAGAGGGGGAAG-3′

IL5	Forward 5′-TAGCTCTTGGAGCTGCCTACGTGTAT-3′ Reverse 5′-AAGCAGTGCCAAGGTCTCTTTCAC-3′

IL6	Forward 5′-ATTCTGCGCAGCTTTAAGGA-3′ Reverse 5′-AACAACAATCTGAGGTGCCC-3′

IL10	Forward 5′-CAAGGACTCCTTTAACAACAAGTT-3′ Reverse 5′-GAGATGCCTTCAGCAGAGTG-3′

IL12	Forward 5′-GGAGTACCCTGACACCTG-3′ Reverse 5′-AGATGACCGTGGCTGAGG-3′

IL13	Forward 5′-CCAGAAGGCTCCGCTCTGCAA-3′ Reverse 5′-GTGCGGGCAGAATCCGCTCA-3′

IL17A	Forward 5′-TCACAATCCCACGAAATCCAG-3′ Reverse 5′-GTGAGGTGGATCGGTTGTAG-3′

TGF-*β*	Forward 5′-GGCCAGATCCTGTCCAAGC-3′ Reverse 5′-GTGGGTTTCCACCATTAGCAC-3′

TNF-*α*	Forward 5′-CGAGTCTGGGCAGGTCTACTTT-3′ Reverse 5′-AAGCTGTAGGCCCCAGTGAGTT-3′

IFN-*γ*	Forward 5′-ATGAAATATACAAGTTATATCTTGG-3′ Reverse 5′-TTACTGGGATGCTCTTCGAC-3′

G-CSF	Forward 5′-GAGCAAGTGAGGAAGATCCAG-3′ Reverse 5′-CAGCTTGTAGGTGGCACACTC-3′

IL-17RA	Forward 5′-CCCAGTAATCTCAAATACCACAGTTC-3′ Reverse 5′-CGATGAGTGTGATGAGGCCATA-3′

IL22	Forward 5′-TTGAGGTGTCCAACTTCCAGCA-3′ Reverse 5′-AGCCGGACGTCTGTGTTGTTA-3′

IL23	Forward 5′-CGTCTCCTTCTCCGCTTCAA-3′ Reverse 5′-ACCCGGGCGGCTACAG-3′

NFKB	Forward 5′-CACTGCTCAGGTCCACTGTC-3′ Reverse 5′-CTGTCACTATCCCGGAGTTCA-3′

STAT3	Forward 5′-GAGGACTGAGCATCGAGCA-3′ Reverse5′-CATGTGATCTGACACCCTGAA-3′

CCR5	Forward 5′-CAAAAAGAAGGTCTTCATTACACC-3′ Reverse 5′-CCTGTGCCTCTTCTTCTCATTTCG-3′

CX3CL1	Forward 5′-GGATGCAGCCTCACAGTCCTTAC-3′ Reverse 5′-GGCCTCAGGGTCCAAAGACA-3′

CXCL5	Forward 5′-TGGACGGTGGAAACAAGG-3′ Reverse 5′-CTTCCCTGGGTTCAGAGAC-3′

CXCL12	Forward 5′-TCAGCCTGAGCTACAGATGC-3′ Reverse 5′-CTTTAGCTTCGGGTCAATGC-3′

**Table 2:** Flow cytometry results of progenitor cells.

	Myometrium	Leiomyoma
HLA-DR	−	−
CD14	−	−
CD19	−	−
CD34	−	−
CD45	−	−
CD73	+	+
CD90	+	+
CD105	+	+
CD9	−	−

Positive immunolabelling (+) was defined as a level of fluorescence > 90% of the corresponding isotype-matched control antibodies. Percentages < 2% were considered negative (−). No statistically significant differences were found among the twelve cultures.
